# Local Structure and Dynamics in MPt(CN)_6_ Prussian Blue Analogues

**DOI:** 10.1021/acs.chemmater.4c01013

**Published:** 2024-05-30

**Authors:** Elodie A. Harbourne, Helena Barker, Quentin Guéroult, John Cattermull, Liam A. V. Nagle-Cocco, Nikolaj Roth, John S. O. Evans, David A. Keen, Andrew L. Goodwin

**Affiliations:** † Department of Chemistry, University of Oxford, Inorganic Chemistry Laboratory, South Parks Road, Oxford OX1 3QR, U.K.; ‡ Department of Materials, University of Oxford, Parks Road, Oxford OX1 3PH, U.K.; § Cavendish Laboratory, University of Cambridge, JJ Thompson Avenue, Cambridge CB3 0HE, U.K.; ∥ Department of Chemistry, Durham University, Durham DH1 3LE, U.K.; ⊥ ISIS Facility, Rutherford Appleton Laboratory, Harwell Campus, Didcot OX11 0QX, U.K.

## Abstract

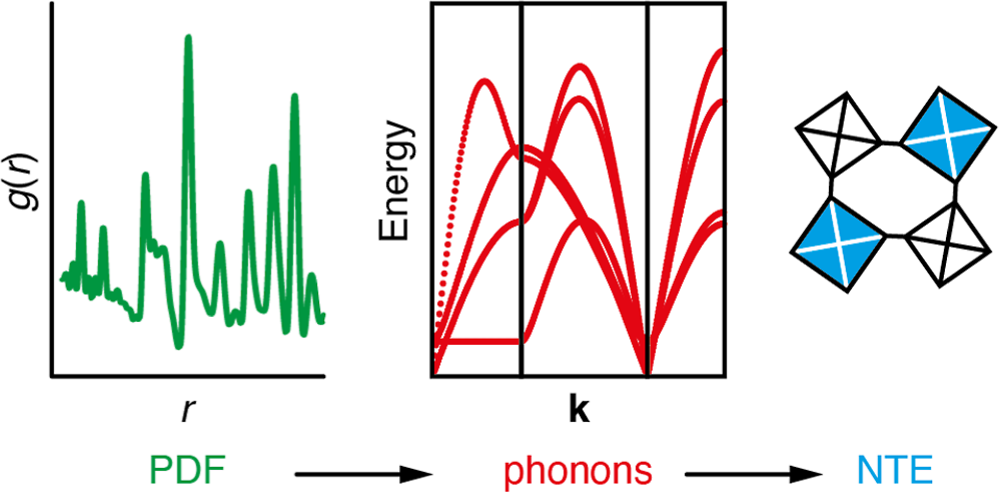

We use a combination of X-ray pair distribution function (PDF) measurements, lattice dynamical calculations, and *ab initio* density functional theory (DFT) calculations to study the local structure and dynamics in various MPt(CN)_6_ Prussian blue analogues. In order to link directly the local distortions captured by the PDF with the lattice dynamics of this family, we develop and apply a new “interaction-space” PDF refinement approach. This approach yields effective harmonic force constants, from which the (experiment-derived) low-energy phonon dispersion relations can be approximated. Calculation of the corresponding Grüneisen parameters allows us to identify the key modes responsible for negative thermal expansion (NTE) as arising from correlated tilts of coordination octahedra. We compare our results against the phonon dispersion relations determined using DFT calculations, which identify the same NTE mechanism.

## Introduction

**Figure 1:**
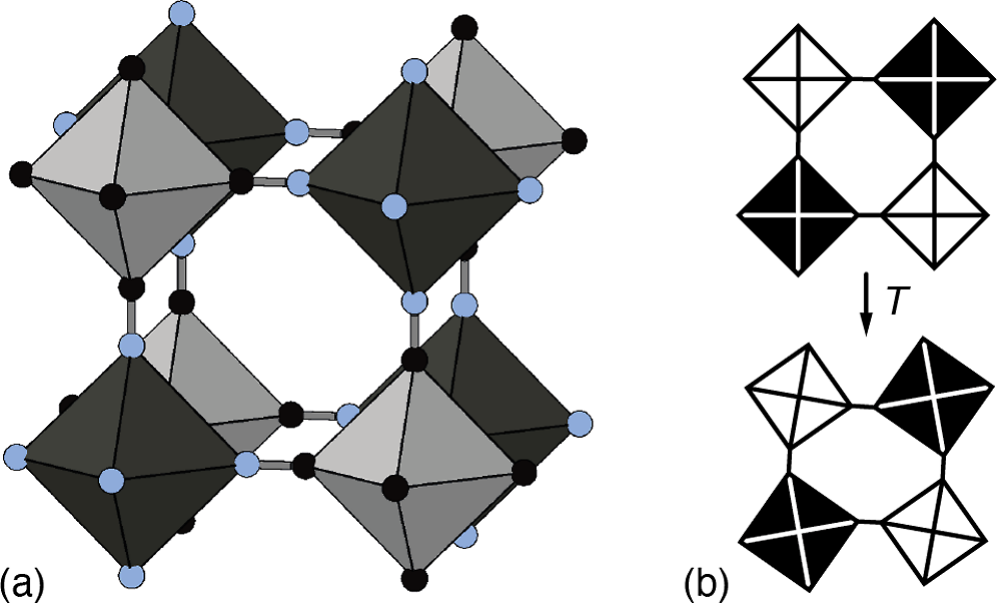
(a) Representation of the basic structure of transition-metal hexacyanoplatinate PBAs.^2^ M^2+^ and Pt^4+^ coordination environments are shown as dark and light octahedra, respectively; C and N atoms are shown as black and blue spheres. (b) The mechanism for NTE typically proposed for this family involves thermally driven correlated rotations of neighboring octahedra, which act to fold the structure in on itself as temperature is increased.^4,5^

Transition-metal hexacyanoplatinates, M^II^Pt^IV^(CN)_6_, are a compositionally simple subfamily of the broader family of Prussian blue analogues (PBAs) A_*x*_M[M′(CN)_6_]_*y*_·*z*H_2_O^1−3^ of particular interest for their negative thermal expansion (NTE) behavior.^4,5^ Their structures are assembled from a simple cubic network of octahedrally coordinated M^2+^ and Pt^4+^ ions bridged by linear Pt–CN–M linkages [Figure 1a]. Whereas many PBAs harbor a variety of different kinds of structural complexity,^6^ including hexacyanometallate vacancies,^7^ tilt instabilities,^8^ and correlated Jahn–Teller (JT) disorder,^9^ the hexacyanoplatinates are structurally much simpler and effectively defect-free.^5^ As a consequence, their local structures are characterized by thermal (phonon-driven) displacements away from the average structure, rather than by occupational or static disorder of any kind. Hence, one expects a direct relationship between the local structure and dynamics in the hexacyanoplatinates.

Surprisingly little is known about the lattice dynamics of this family, despite the observation of widespread NTE among its members.^4,5,10−13^ NTE—the phenomenon of contraction on heating—is usually driven by either electronic or phonon mechanisms;^14^ it is the latter that is relevant to the hexacyanoplatinates.^4,5,15,16^ Formally, phonon-driven NTE requires a material to support phonon modes with negative Grüneisen parameters.^17,18^ For a given phonon branch ν at wave-vector **k**, its Grüneisen parameter
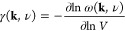
1captures the dependence of the phonon frequency ω on changes in volume *V*.^19,20^ A value γ ≃ 1 is typical of conventional materials, and signifies that phonon frequencies increase under pressure. By contrast, modes responsible for NTE have γ < 0—i.e., their frequencies decrease as volume decreases—which is why the material can increase its vibrational entropy by reducing volume. Phonon-driven NTE is most extreme in cases where NTE modes have large and negative Grüneisen parameters and at the same time are low in energy (i.e., rapidly populated thermally). It is generally assumed that the key NTE phonons in hexacyanoplatinates—and PBAs more generally—involve correlated rotations of MN_6_ and PtC_6_ octahedra [Figure 1b] but this hypothesis remains to be tested.^4,5,10−13^

From an experimental perspective, it is known that changing the radius of M^2+^ cations allows tuning of the magnitude of NTE in hexacyanoplatinates.^5^ Larger cations drive stronger NTE, which is understood in general terms as a consequence of the weakening of M–N interactions (lowering phonon frequencies and amplifying their thermal populations). It is also known that introducing water molecules within the cavities of the hexacyanoplatinate framework structure dampens NTE modes, even causing a switch from negative to positive thermal expansion in the case of ZnPt(CN)_6_·*x*H_2_O.^4^ Hence, NTE phonons likely involve displacements into the ordinarily open space of the cubic cavity. Phonon dispersion curves and the corresponding Grüneisen parameters have been calculated for the closely related material ScCo(CN)_6_ in ref (13). In that study, the strongest NTE modes are indeed associated with correlated octahedral tilts; however, the phonon relations were not corrected for the choice of unconventional unit-cell setting, which complicates their interpretation. We have also flagged elsewhere the potential for translational “shift” distortions to contribute to NTE, but the importance of this contribution is unknown.^21^

It was in this context that we were interested in understanding the local structure of transition-metal hexacyanoplatinates: after all, the distortions away from their average structure are dominated by those phonon modes responsible for NTE. An excellent experimental handle on local structure is provided by pair distribution function (PDF) measurements.^22,23^ The PDF represents a histogram of interatomic distances, weighted by the corresponding atomic concentrations and scattering strengths. X-ray PDF measurements have been used previously to provide insight into local structure in PBAs,^24−27^ but their quantitative interpretation is particularly difficult. This difficulty arises because standard PDF analysis methods assume that the degree of correlation in pairwise displacements decreases monotonically with increasing distance, but in PBAs atom-pairs at similar distances can be correlated to very different extents (e.g., inter- vs intrapolyhedral combinations).

Consequently, we develop here a modified “interaction-space” approach^28,29^ for interpreting the PDFs of MPt(CN)_6_ whereby we optimize the parameters of an intentionally simplistic empirical potential model so as to reproduce the experimentally determined pair correlations. In spirit, our approach shares much in common with the empirical potential structure refinement methodology used to interpret the PDFs of heavily disordered materials such as liquids and glasses.^30^ In the context of understanding NTE in hexacyanoplatinates, a particular advantage of determining effective potentials that govern their lattice dynamics is that we are able to identify directly the likely phonon modes most important in driving NTE. An interesting corollary is that we obtain this insight even by fitting data collected at a single (ambient) temperature, rather than relying on decoding the thermal evolution of specific distortion patterns as is customary in the field.^31,32^

Anticipating our results, we will come to show that the experimental PDFs of MnPt(CN)_6_ and CuPt(CN)_6_ can be well described using a very simple potential with strong intrapolyhedral force constants and weak interpolyhedral angular force constants. The specific parametrizations obtained by fitting to the PDFs allow determination of simplified phonon dispersion relations, which do indeed identify correlated tilt modes as the key microscopic driving forces for NTE. Density functional theory (DFT) calculations carried out for the closely related closed-shell analogue ZnPt(CN)_6_ reflect the same key NTE phonon branches, suggesting a universal NTE mechanism in this family.

## Methods

### Synthesis

We prepared polycrystalline samples of MnPt(CN)_6_ and CuPt(CN)_6_ following the procedure of ref (33). An aqueous solution of M(SO_4_)_2_·*x*H_2_O (0.5 mmol in 0.5 mL H_2_O) was added dropwise with stirring to an aqueous solution of K_2_Pt(CN)_6_ (0.5 mmol in 0.5 mL H_2_O). Pale crystalline powders of MPt(CN)_6_·*x*H_2_O appeared in solution over a matter of minutes, and the solids were isolated by filtration. The solids were dehydrated by heating under vacuum for 24 h at 70 °C to afford ∼100 mg each of white polycrystalline MnPt(CN)_6_ and pale-green polycrystalline CuPt(CN)_6_.

### X-ray PDF Measurements

X-ray total scattering measurements were performed using the I15-1 (XPDF) beamline at the Diamond Light Source. Samples were loaded in thin-walled borosilicate capillaries (1 mm diameter) and data measured under ambient conditions with an exposure time of 600 s. The X-ray energy used was 76.69 keV (λ = 0.161669 Å), which gave a useable scattering range 0.35 ≤ *Q* ≤ 19 Å^–1^. The data were corrected for background, multiple scattering, container scattering, Compton scattering, absorption, and Bremsstrahlung effects and placed on an absolute scale using GudrunX.^34^ The normalized data were Fourier transformed to yield the corresponding PDFs, following the procedure outlined in refs (34 and 35).

### Interaction-Space PDF Refinements

We used an iterative approach to refine the parameters of an empirical lattice dynamical model against the experimental PDF data. We will describe the potential model itself in detail in the Results section below. Starting from a sensible estimate of the relevant interatomic force constants, we used a Monte Carlo (MC) algorithm to generate an atomistic representation of the corresponding MPt(CN)_6_ structure that included thermal fluctuations. Our MC simulations employed a 6 × 6 × 6 supercell of the parent *Fm*3̅*m* (or *F*4/*mmm*,^a^ M = Cu) unit-cell and hence contained 12,096 atoms in total. The simulations were carried out using a MC temperature of 300 K and were allowed to proceed until convergence was achieved.

The MC configuration was then used as input for a constrained PDF + Bragg scattering refinement carried out using the TOPAS software.^36^ In this refinement, the positions of atoms were fixed and the corresponding PDF calculated as described in ref (37); all comparisons were made over the real-space range 1 < *r* < 30 Å, using a bin-width of 0.02 Å. The configurational average of this structural description was determined and used to calculate a corresponding fit to the scattering function (Bragg intensities) in reciprocal space. Atomic displacement parameters were fixed at a constant value very much smaller than that associated either with the real-space resolution at *Q*_max_ = 19 Å^–1^ or with the distribution of atomic sites in the collapsed (average) structure. Unit-cell dimensions, peak–shape parameters, and background functions were collectively refined against the 1 ≤ 2θ ≤ 16° angular range of our experimental Bragg data. The resulting goodness-of-fit represented the simultaneous quality of fits to both real- and reciprocal-space data, weighted as a simple linear sum of the corresponding *R*_wp_ values.

The iterative nature of our approach then involved varying the values of each individual empirical parameter one-by-one, repeating the MC simulations using these adjusted values, and subsequent fitting to the PDF and Bragg data. The resulting fit qualities were monitored in order to determine the set of parameters that gave the best global fit-to-data. Further details of this approach and the specific refinement parameters employed are given in the Supporting Information.

### Harmonic Lattice Dynamical Calculations

The GULP lattice dynamical code,^38,39^ driven using the empirical potential determined from our PDF measurements, was used to obtain phonon dispersion relations for MnPt(CN)_6_. These calculations were carried out following structure optimization and were evaluated at intervals of 0.05 reciprocal lattice units along high-symmetry directions of the Brillouin zone. Our GULP parametrization took into account the difference in force constants that arises by excluding mass in MC simulations driven by eq 2. Grüneisen parameters were determined in the quasiharmonic regime by repeating phonon calculations at fixed volumes, respectively, 1% greater and 1% lower than the equilibrium cell volume. Eigenvector matching was used to track phonon frequency variations and the corresponding values of γ were calculated according to eq 1.

### DFT Calculations

*Ab initio* DFT calculations were carried out using the CASTEP program, version 16.11.^40^ To avoid open-shell configurations, we focused our calculations on the closed-shell analogue ZnPt(CN)_6_. Ultrasoft pseudopotentials supplied as part of the CASTEP package were used together with the standard PBEsol generalized-gradient approximation. The integration of electronic states was performed using a Monkhorst–Pack grid of 3 × 3 × 3 wave vectors and a plane-wave cutoff energy of 700 eV.

All the structure geometries were initially optimized using the BFGS method with convergence of energies to 10^–10^ eV per atom and convergence of force to 10^–6^ eV Å^–1^.

For the calculation of the phonon dispersion relations, CASTEP single point calculations were used in conjunction with Phonopy.^41,42^ Phonopy’s finite displacement method was used with a 2 × 2 × 2 supercell. Single point calculations were performed on multiple supercells with different displacements to then calculate a force-constant matrix. Phonon dispersions were then plotted using intervals of 0.05 reciprocal lattice units along high-symmetry directions of the Brillouin zone. Grüneisen parameters were determined in the quasiharmonic regime by repeating phonon calculations at fixed volumes, respectively, 1% greater and 1% lower than the equilibrium cell volume. Eigenvector matching was used to track phonon frequency variations and the corresponding values of γ were calculated according to eq 1. The neutron-weighted phonon density of states was determined from our force-constant matrix outputted from CASTEP using the QpointPhononModes module in Euphonic.^43^ A Monkhorst–Pack grid of 21 × 21 × 21 was used to sample reciprocal space, with an energy broadening of 0.6 meV, matching that of the experimental data of ref (44).

## Results and Discussion

### X-ray Pair Distribution Functions

**Figure 2:**
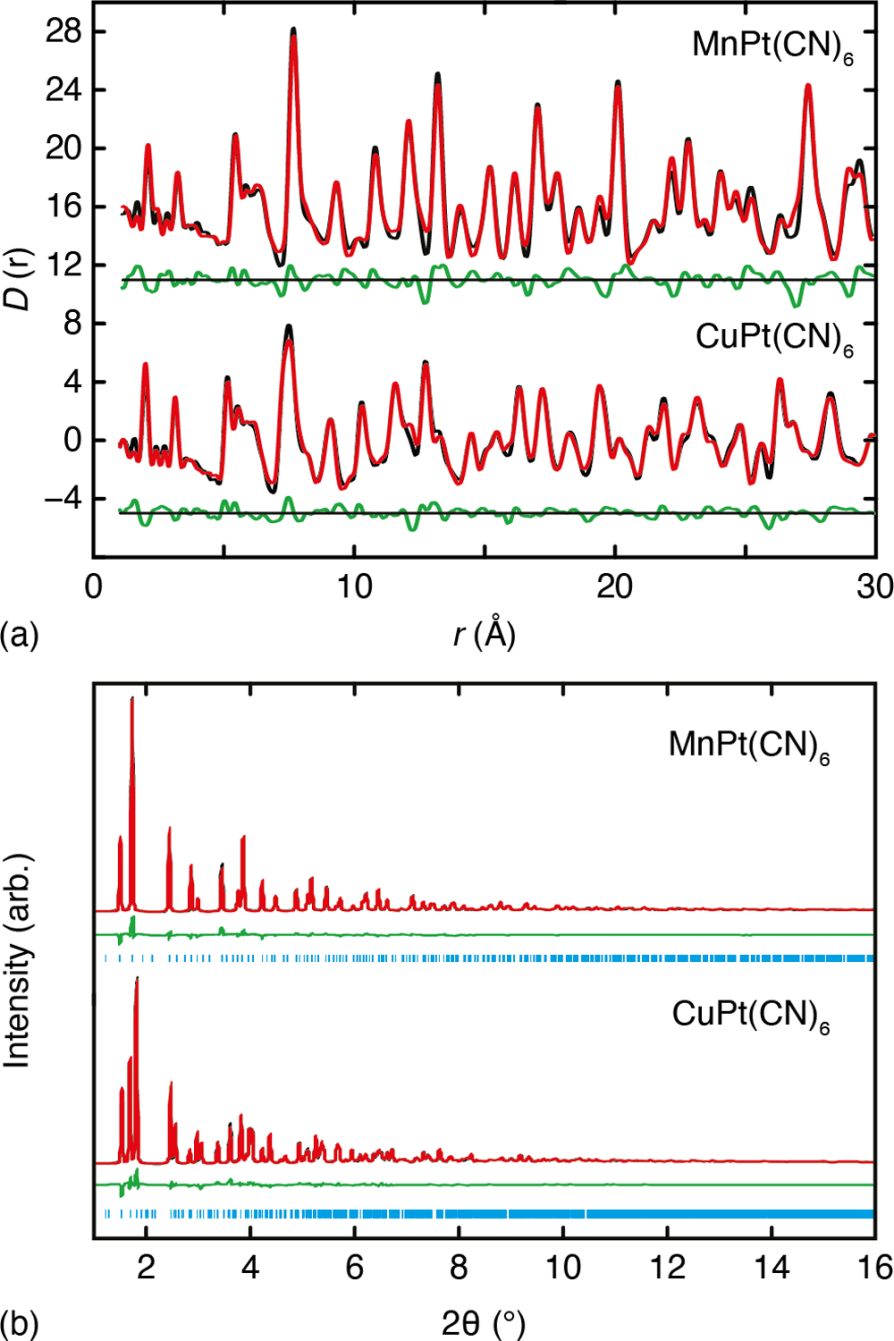
Experimental X-ray total scattering data and empirical MC fits. Panel (a) shows the X-ray PDFs for MnPt(CN)_6_ and CuPt(CN)_6_. Data are shown in black, the fit shown in red, and the difference function (data–fit) shown as a green line, shifted vertically beneath the data by 5 units. Panel (b) shows the corresponding reciprocal-space Bragg diffraction pattern fits obtained from the same MC configurations. Colors are as in (a), with calculated reflection positions shown as blue tick marks.

The experimental X-ray pair distribution functions, measured under ambient conditions for our samples of MnPt(CN)_6_ and CuPt(CN)_6_ are shown in Figure 2a. In both cases, the strongest features in the PDFs tend to involve contributions from pairwise interactions involving Pt. For example, we cannot clearly resolve the cyanide C–N pair correlation at 1.15 Å, but the PDFs place strong constraints on this distance nonetheless via the Pt–C/M–N and Pt–N/M–C peaks at ∼2.0 and 3.1 Å, respectively. The amplitude of the PDF oscillations is reduced in the case of CuPt(CN)_6_. This is because long-range cooperative JT order reduces the crystal symmetry from *Fm*3̅*m* to *F*4/*mmm* and hence splits the positions of otherwise-equivalent pair correlations, giving broader peaks in the PDF. The corresponding powder X-ray diffraction patterns—i.e., the reciprocal-space data from which the PDFs are derived—are shown in Figure 2b. The presence of sharp Bragg reflections for both samples is characteristic of highly crystalline samples.

We initially attempted to fit these data making use of the “real-space Rietveld” approach implemented in TOPAS, which uses similar PDF refinement strategies to those performed by, e.g., PDFGui.^37,45^ In doing so, we encountered the difficulty that PDF peak widths could not be accounted for satisfactorily. The key obstacle was that, in the experimental PDF, peak widths do not increase monotonically with increasing separation *r*. This effect is characteristic of varying interaction strengths operating over common length scales (e.g., inter- vs intrapolyhedral atom-pairs). In such cases, one commonly used solution is to employ “big-box” modeling techniques, such as reverse Monte Carlo (RMC),^46−48^ which allow atomic coordinates in supercell configurations to refine in order to best fit the PDF. Such configurations can then capture the mixture of different pair correlation widths that may be present. One important limitation of such approaches is the very large number of parameters involved. Because our scientific focus is on understanding the underlying dynamical behavior of hexacyanometallates, we devised a different approach based on empirical potentials.

Our starting point was to assume that the key degrees of freedom accessible to hexacyanometallates involve stretching of Pt–C/M–N bonds (the C–N bond itself being essentially rigid at ambient temperature) and also variation in the intra- and interpolyhedral angles. In this way, we write the lattice energy for MnPt(CN)_6_ as the sum
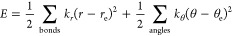
2The first term involves a sum over the two types of deformable bonds, each of which carries its own bond strength *k*_*r*_ and equilibrium distance *r*_e_. Similarly, the second term represents a sum over contributions from three types of angles: we consider the (right-angled) C–Pt–C, N–Mn–N, and (linear) Mn–N–C triplets to be deformable, but in order to limit the number of variables treat the Pt–C–N linkage as stiff. This approximation is justified by the relatively high bending force-constants observed among hexacyanoplatinates.^3^ The equilibrium angles θ_e_ are taken as either  or π as appropriate. This model has seven independent parameters, to which we add an eighth—namely, the length of the (rigid) C–N bond. The values of these parameters as determined from our fits to data are listed in Table 1.

**Table 1:** Empirical Parameters Used to Model the X-ray PDFs of MPt(CN)_6_ (M = Mn,Cu), Together with Their Refined Values^a^

parameter	MnPt(CN)_6_	CuPt(CN)_6_
*k*_*r*_(Pt–C) (eV/Å^2^)	9.4	8.5
*k*_*r*_(M–N) (eV/Å^2^)	3.5	15.5 (eq.)
		0.6 (ax.)
*r*_e_(Pt–C) (Å)	2.02	2.01
*r*_e_(M–N) (Å)	2.32	2.08 (eq.)
		2.33 (ax.)
*k*_θ_(C–Pt–C) (eV/rad^2^)	14.1	4.3
*k*_θ_(N–M–N) (eV/rad^2^)	0.74	0.85 (eq./eq.)
		0.68 (eq./ax.)
*k*_θ_(M–N–C) (eV/rad^2^)	0.02	0.08 (eq.)
		0.01 (ax.)
*r*(C–N) (Å)	1.14	1.15

a For CuPt(CN)_6_, terms involving axial (ax.) and/or equatorial (eq.) Cu–N linkages are distinguished accordingly.

The case of CuPt(CN)_6_ is more complicated as a consequence of the JT distortion of the Cu^2+^ coordination environment. In our model for this system, we now distinguish the stretching force constants and equilibrium distances for axial and equatorial Cu–N links, as we do the various different angular terms involving Cu–N bonds. These additional considerations increase the number of free variables to 12 for CuPt(CN)_6_.

For a given set of force-constant and equilibrium–distance parameters, we used MC simulations, carried out at ambient temperature, to obtain atomistic representations of the structures of MnPt(CN)_6_ and CuPt(CN)_6_. In these atomistic configurations, the displacements of atoms away from their high-symmetry positions are entirely characteristic of the energetics of the bond and angle deformations governed by eq 2. The PDFs and powder diffraction peak intensities associated with each model can be calculated directly from the atom coordinates within the corresponding supercell. These calculated scattering functions can be compared against our experimental data and a goodness-of-fit determined. By tracking the fit quality as a function of each individual parameter, we can refine iteratively the values of these parameters and hence converge on an experiment-driven parametrization of eq 2. We found that the experimental data showed differing levels of sensitivity to different parameters and further discussion of this point is given in the Supporting Information.

**Figure 3:**
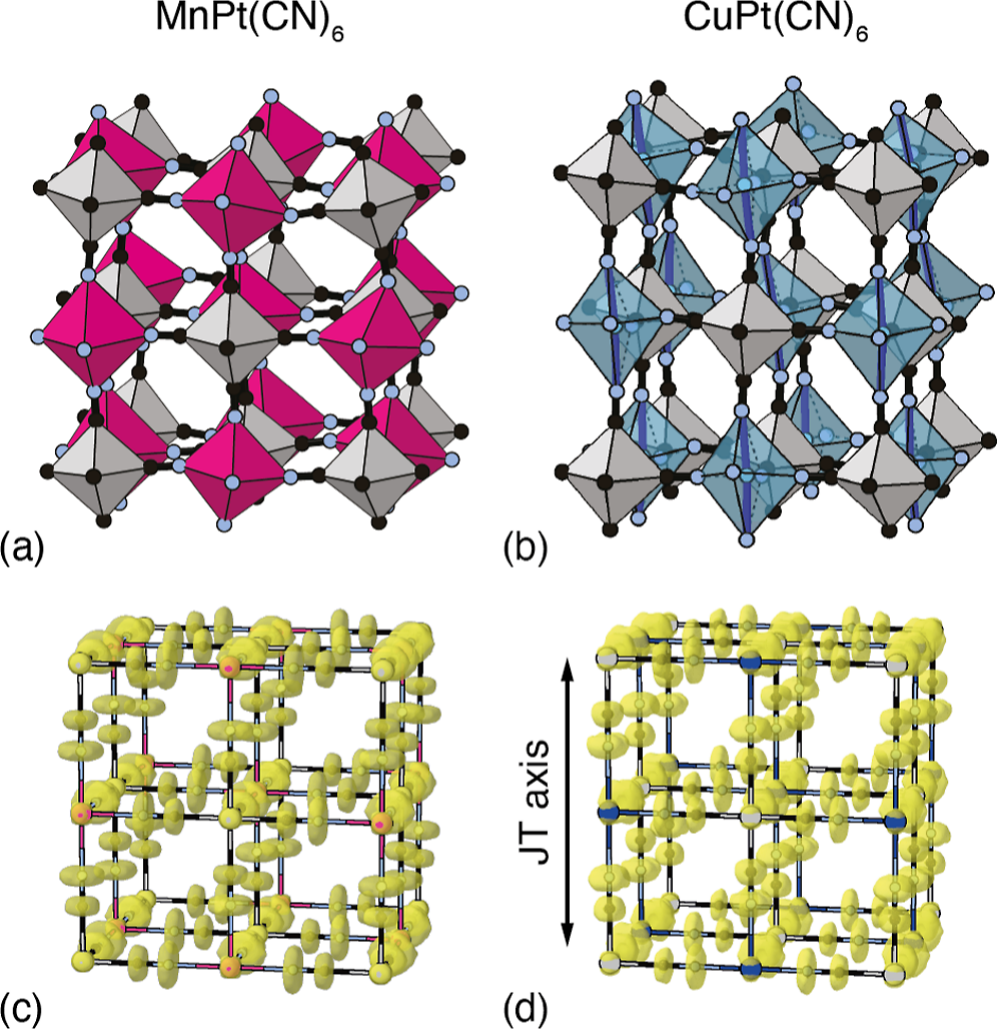
Representations of the local and average structures of MPt(CN)_6_ PBAs determined in our PDF refinements. Panels (a,b) show small fragments from MnPt(CN)_6_ and CuPt(CN)_6_ configurations. Various bond-length and bond-angle distortions are evident and these should be interpreted as snapshots of thermal fluctuations through vibrational motion. Atom colors as in Figure 1a, with M = Mn and Cu shown in pink and blue, respectively. Panels (c,d) show the collapsed configurations from which the Bragg intensities were calculated. The yellow surfaces represent the volume occupied by 98% of sites around each atom position. In both cases, the corresponding crystal symmetry has been applied, and we use the unconventional face-centered tetragonal cell for CuPt(CN)_6_ to aid comparison between the two structures. The direction of the JT axis is indicated by an arrow.

The fits-to-data obtained using this “interaction-space” refinement approach are shown in Figure 2 and the corresponding parameters are given in Table 1. The variation in parameters for common interactions in both hexacyanoplatinates gives a sense for the corresponding degree of uncertainty (which can be large). An important distinction from conventional PDF refinements is that, in our case, the PDF and Bragg diffraction intensities for a given system are calculated from one and the same model. Representative fragments of such models are shown in Figure 3a,b. Because X-ray PDF measurements are energy-integrated, these configurations can be interpreted as instantaneous snapshots of the corresponding systems undergoing vibrational motion.^22^ The empirical parameters themselves show reasonable trends: deformations of PtC_6_ octahedra are more energetically expensive than those of MN_6_ octahedra, and the axial Cu–N linkage is associated with weaker interactions, as expected.^33^

The corresponding average structures, obtained by collapsing the supercell onto a single unit-cell and subsequently applying the appropriate space-group symmetry (*Fm*3̅*m* or *F*4/*mmm*), are shown in Figure 3c,d. From these models, it is clear that the Mn-containing system is more dynamic than the Cu-containing system. This observation is consistent with the stronger NTE behavior of MnPt(CN)_6_ relative to CuPt(CN)_6_.^5^ What is also clear is why conventional PDF refinements using monotonic *r*-dependent peak widths fail: the distributions around M and Pt positions are very tightly bunched (and hence the corresponding PDF peaks very narrow), while the C/N distributions are very diffuse (and hence any C/N–C/N PDF peaks will be broader). Note also the anisotropy of the Cu^2+^ distributions: displacements along the JT axis are clearly much larger than those in a perpendicular direction. Through cooperative displacements, this anisotropy leaves a small imprint also on the Pt^4+^ distribution, despite the six Pt–C bonds being governed by the same effective interaction.

### Local Structure

**Figure 4:**
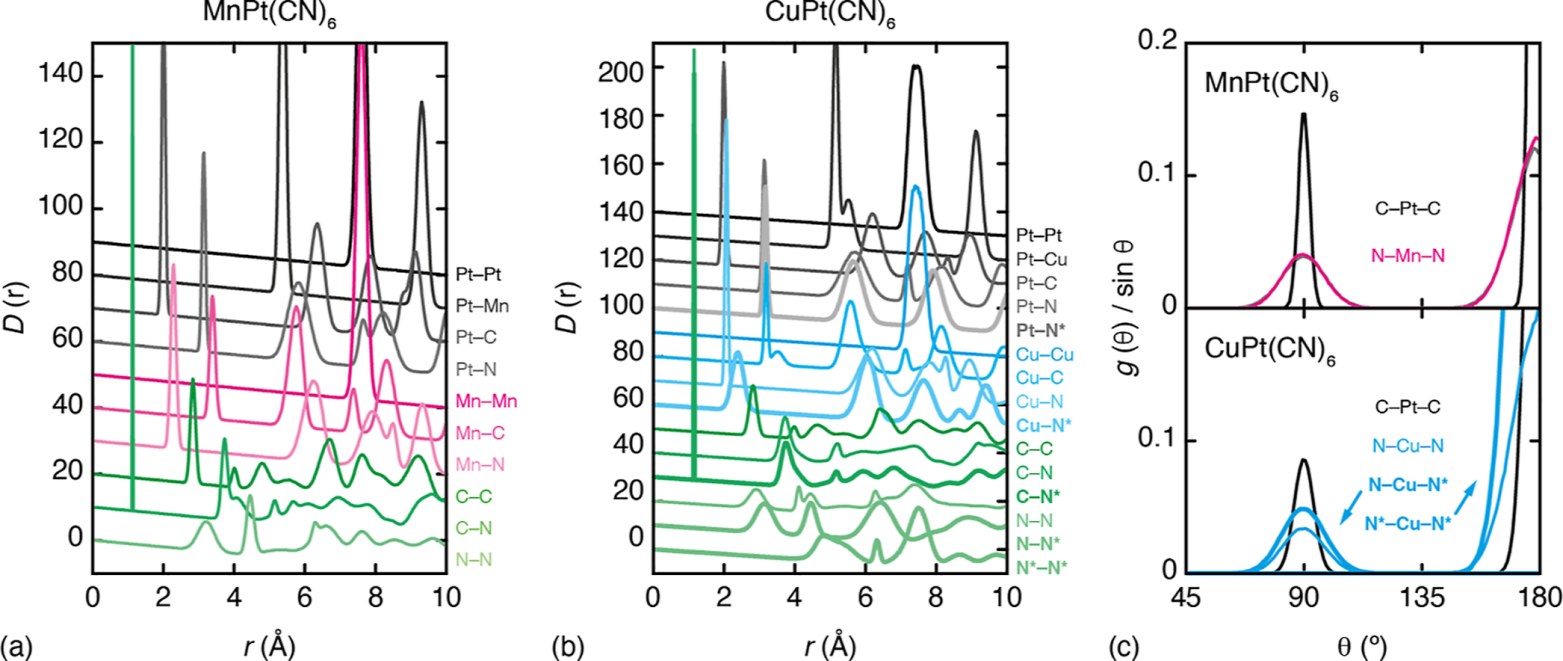
Partial pair-correlation functions for (a) MnPt(CN)_6_ and (b) CuPt(CN)_6_ extracted from our PDF-driven MC simulations. Successive curves are shifted vertically by 10 units. Correlations involving N atoms along local JT axes of Cu^2+^ coordination environments are highlighted by an asterisk and displayed using bolder lines. (c) The intrapolyhedral angle distributions within PtC_6_ and MN_6_ octahedra, highlighting the additional angular flexibility along the JT axis in (Cu^2+^) configurations.

In order to interrogate further the local structural features captured by our PDF-derived MC configurations, we extracted key pair and triplet correlation functions. These are shown in Figure 4 and, by virtue of our fitting process, should be interpreted as the correlation functions deconvolved of instrumental and experimental broadening. Our expectations based on the magnitude of the various empirical interaction parameters are borne out in practice in terms of the corresponding correlations. We see a mixture of broad and sharp features for different pair correlations at similar separations. The presence of a JT distortion in the Cu^2+^ coordination environment is clear in the difference in nearest-neighbor Cu–N pair correlation for axial and equatorial pairs. In Figure 4c, we show the distribution of intrapolyhedral angles for the PtC_6_, MnN_6_, and CuN_6_ octahedra. In the latter case, we can decompose the distribution into those components from axial–axial, axial–equatorial, and equatorial–equatorial N–Cu–N triplets. What emerges is that the in-plane CuN_4_ geometry is relatively tightly constrained, but that there is much greater flexibility along the JT axis. Cooperative JT distortion also broadens the Cu–Cu, Cu–Pt, and Pt–Pt pair correlations, which is why the PDF oscillation amplitudes are smaller in magnitude for CuPt(CN)_6_ than MnPt(CN)_6_ at high *r*.

From a methodological perspective, we note that by using a harmonic parametrization to fit the X-ray PDFs, the various pair correlations necessarily adopt smooth forms that are approximately Gaussian in shape. This contrasts the situation in other big-box (e.g., RMC) refinements, where small fluctuations in the data—especially at low distances—can translate to unphysical features in the partial pair correlation functions, such as spikes and truncation peaks.^48^

### Lattice Dynamical Calculations

**Figure 5:**
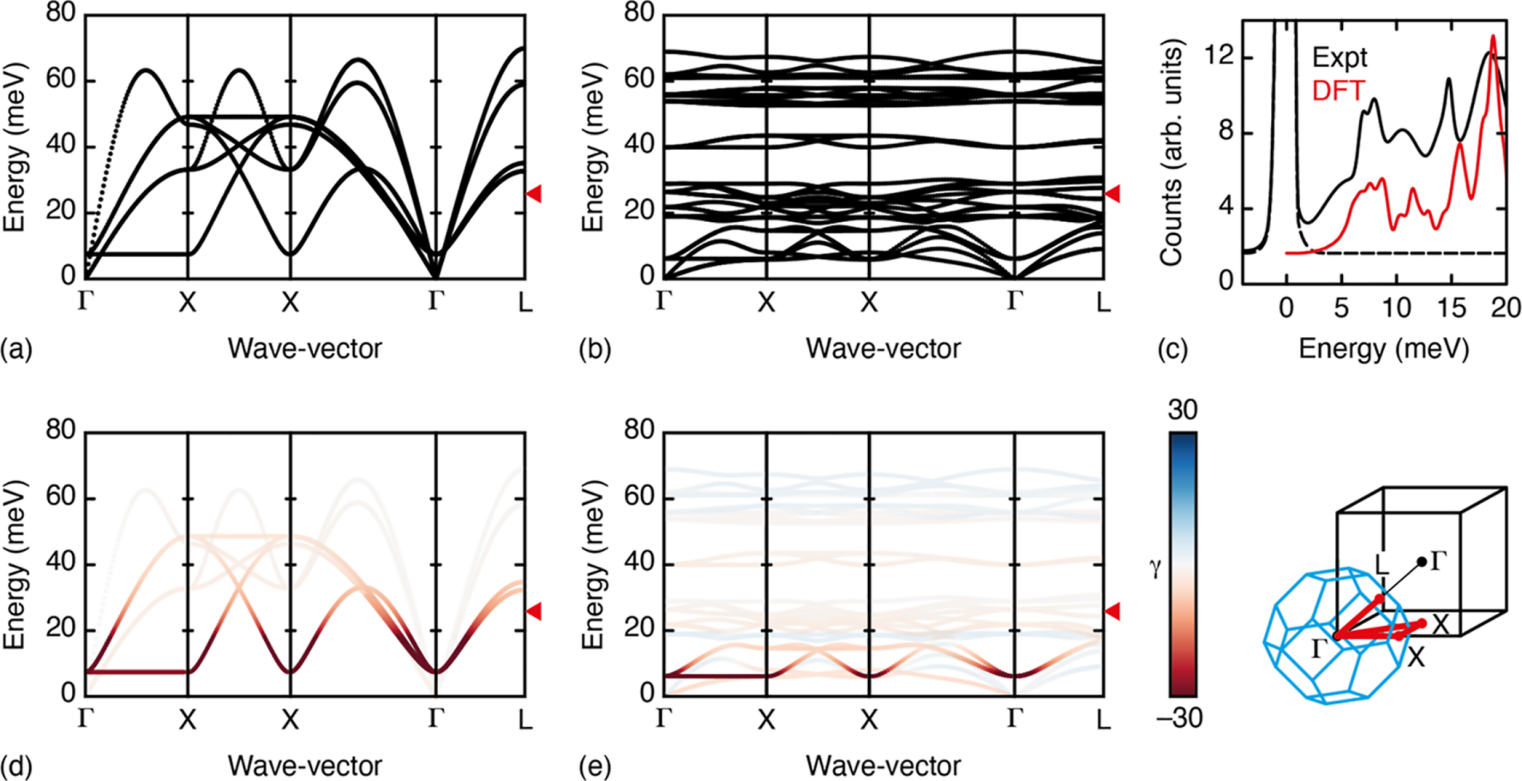
Lattice dynamics in MPt(CN)_6_ PBAs. (a) Simplified low-energy phonon dispersion relation for MnPt(CN)_6_ obtained from the empirical lattice-dynamical model used in our PDF fits. Note the presence of a dispersionless branch at ∼8 meV along the Γ–X direction, which corresponds to correlated octahedral tilts as shown in Figure 1b. Thermal energy at 300 K is indicated by a small triangular mark on the right-hand side of the dispersion curves. (b) The all-atom low-energy phonon dispersion of ZnPt(CN)_6_ determined using DFT. The same dispersionless branch along Γ–X is present, but among the many differences from the curves in (a) is a dramatic softening of the transverse acoustic branches, associated with cooperative shift displacements.^21,49^ (c) Comparison of the neutron-weighted phonon density of states in ZnPt(CN)_6_ between experiment (ref (44)) and our DFT calculations. Vertical scales are arbitrary; the key observation is that the energy scales (horizontal peak positions) are well matched between the two. Panels (d,e) show the same phonon dispersion curves given in (a,b), but with branches colored according to the corresponding mode Grüneisen parameters. The key NTE modes (γ ≪ 0; deep red) are identified in both cases as the cooperative octahedral tilts. The reciprocal-space path used in our phonon representations is shown schematically on the right-hand side as the bold red trace within and beyond the first Brillouin zone (blue cuboctahedron). Experimental data in panel (c) are reprinted from Physica B: Condensed Matter, volume 385, K. W. Chapman, M. Hagen, C. J. Kepert, P. Manuel, Low energy phonons in the NTE compounds Zn(CN)_2_ and ZnPt(CN)_6_, pp. 60–62, Copyright (2006), with permission from Elsevier.

A key advantage of interpreting our experimental PDF measurements in this way is that we are able to calculate directly from eq 2 the corresponding phonon dispersion curves and hence quantify the extent to which particular vibrational modes are dominating atomic displacements at ambient temperature. The low-energy phonon dispersion calculated in this way for MnPt(CN)_6_ is shown in Figure 5a; the equivalent result for CuPt(CN)_6_, which is more complicated as a consequence of the lower crystal symmetry, is given and discussed in the Supporting Information. A key feature of the low-energy phonon spectrum is a dispersionless branch that runs along the **k** = [00ξ] direction from Γ to X. Inspection of the corresponding mode eigenvectors identifies this branch as the conventional octahedral tilt distortion, with the tilt axis parallel to **k**. This is the same low-energy branch that would ordinarily extend along the Brillouin zone boundary from M (in-phase tilts) to R (out-of-phase tilts) in the conventional (primitive cubic) perovskite aristotype.^50^

Our DFT calculations for ZnPt(CN)_6_—the closed-shell analogue of MnPt(CN)_6_—give the low-energy dispersion relations shown in Figure 5b; the corresponding neutron-weighted density of states compares favorably with the experimental measurements of ZnPt(CN)_6_ in ref (44), as shown in Figure 5c. At face value, these dispersion curves appear very different from those of our PDF-derived lattice dynamical model. One difference that is easily explained is that the treatment of C–N bonds and Pt–C–N angles as rigid in the empirical model reduces the number of phonon branches by nearly a factor of 2: this is why the DFT result looks more complicated. Indeed, at very low energy, there is an additional set of branches in the DFT dispersion relation that corresponds to the “shift” degree of freedom identified in ref (21) (these become the transverse acoustic modes as **k** → Γ). These distortions require flexing of the Pt–C–N angle which is why they are not present in the empirical dispersion curves. Nevertheless, an important similarity between the two sets of phonon dispersion curves is that the octahedral tilt branch is again dispersionless in the DFT calculations and occurs at a very similar energy to that given by the empirical model.

**Figure 6:**
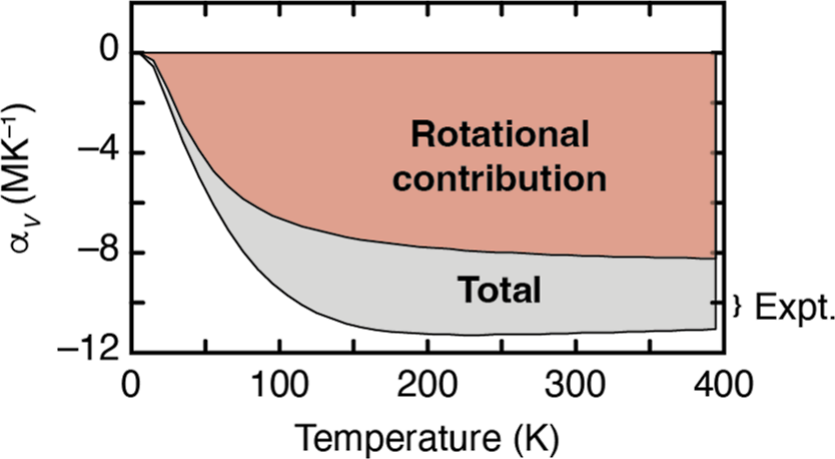
Contribution of correlated rotational vibrations to NTE in ZnPt(CN)_6_ as a function of temperature, determined from our DFT-calculated phonon dispersion relations. The vertical axis gives the volumetric coefficient of thermal expansion α_*V*_, defined as the relative rate of change of volume with respect to temperature.^18^ The range of experimental values reported in refs (4 and 5) are indicated on the right.

In order to make the link to NTE mechanisms, we calculated the Grüneisen parameters for each branch in the phonon spectra. In Figure 5d,e, we show the phonon dispersion curves with intensities and colors weighted by the corresponding values of γ(**k**, ν) with key numerical results listed in Table 2. We expect the DFT result to capture the true NTE mechanism, and what is immediately obvious is that the octahedral tilts along Γ–X are associated with Grüneisen parameters that are particularly large and negative. The columnar shifts also have negative Grüneisen parameters but their magnitudes are sufficiently smaller that the corresponding contribution to NTE is also reduced. We quantify this contribution of rotations to the NTE of ZnPt(CN)_6_ in Figure 6. Importantly, the empirical parametrization also captures precisely this same mechanism—indeed, even the magnitudes of the Grüneisen parameters are qualitatively similar to the DFT result. So, despite the differences in much of the phonon spectra between DFT and empirical calculations, the key distortions at low energies from which NTE arise are actually well matched.

**Table 2:** Mode Grüneisen Parameters for the NTE Tilt Branch in MPt(CN)_6_ Systems at Relevant High-Symmetry Points in the Brillouin Zone

	Γ	*X*
ZnPt(CN)_6_ DFT	–39.4	–37.2
MnPt(CN)_6_ PDF	–27.1	–27.1

The correlated tilt displacement pattern is an example of a rigid-unit mode because it preserves the coordination geometries of both M^2+^ and Pt^4+^ centers.^15,51,52^ Such tilts are pure RUMs only along Γ–X and only for the transverse-polarized branch (i.e., tilts around an axis parallel to **k**). However, common to both PDF- and DFT-derived phonon dispersion relations is the observation that correlated tilts contribute to the NTE even away from this branch—albeit with decreasingly extreme negative Grüneisen parameters. Hence, the slightly distortive quasi-RUMs (QRUMs) play an important role because they extend the reciprocal-space density of NTE modes from one-dimensional lines along specific axes to a volume of space surrounding those lines.^52,53^

An important consideration when interpreting these phonon dispersion relations is the extent to which different branches are populated at ambient temperature. We include in Figure 5 a marker at 26 meV—the available thermal energy at 300 K. Modes at higher energy than this value are relatively unpopulated at the temperature at which our PDF measurements were carried out. Hence, we expect reduced sensitivity to the true energies of this part of the phonon spectrum through PDF analysis. By contrast, the PDF will be dominated by displacement driven by the very lowest-energy branches, which is why the force constants governing octahedral tilts appear to be well constrained in our empirical model. Similar conclusions regarding varying sensitivity of the PDF to contributions from different phonons were reached in the earlier studies of refs (54–56).

## Concluding Remarks

So, what has our analysis achieved? We have shown how a simple model, with remarkably few parameters, is able to describe the key thermal fluctuations in transition-metal hexacyanometallates, accounting for both the experimental PDF and the intensities of Bragg reflections in the X-ray diffraction pattern. The incorporation of the information within PDF data imparts sensitivity to pairwise correlations in these fluctuations, which is what allows us to interrogate the phonon dispersion relations implicit in our empirical model. We find that the low-energy modes—which dominate thermal motion and hence the variation in peak widths in the PDF—are surprisingly well captured in this analysis. In particular, we are able to identify the correct NTE mechanism by inspection of the phonon mode eigenvectors and their corresponding pressure dependence.

The NTE mechanism itself is relatively straightforward: as anticipated elsewhere, the dominant contribution comes from correlated octahedral tilts as shown in Figure 1b. The shift displacements proposed in ref (21) also contribute, but to a much reduced extent. An important subtlety that emerges from our analysis is that cooperative tilts—which are associated with specific lines in reciprocal space—are only able to drive bulk NTE in hexacyanoplatinates because of the contribution of nearby distortive (QRUM) tilts. Hence, the relative deformability of transition-metal coordination environments is an important contributing factor, precisely as implicated in the NTE behavior of other NTE framework materials such as oxides and fluorides.^53,57^ This NTE mechanism may prove to be relatively universal among PBAs,^12^ given the similar conclusions drawn in the DFT study of ScCo(CN)_6_.^13^

Thinking beyond these specific hexacyanoplatinate PBAs, our work also introduces a parameter-efficient methodology for fitting PDF data in systems with competing energy scales. Here, we have intentionally focused on fitting data collected at a single temperature, but our methodology is straightforwardly extended to variable-temperature data sets. In principle, a single set of empirical parameters should account for PDF peak widths across the entire set of data sets, with fits-to-data evaluated using MC simulations performed across a corresponding series of temperatures. This redundancy should improve confidence in the parameter values obtained. In our own work, we have a strong interest in understanding the microscopic behavior of more complex PBAs, including those applied as cathode materials,^58−60^ for which the inclusion of hexacyanometallate vacancies and extra-framework cations significantly complicates the interpretation of the corresponding PDFs.^6^ Our intuition is that sensibly constrained parameter-efficient methodologies to fitting the PDF will be required for robust characterization of the local structure in these more complex cases.

## Supporting Information Available

Details regarding methods, analysis, and supplementary discussion; details of our iterative refinement strategy; results of our local and average structure refinements; and calculated phonon dispersion curves for CuPt(CN)_6_ (PDF)

## Footnotes

a We use the unconventional *F*4/*mmm* setting for CuPt(CN)_6_ in our study so as to simplify comparison to MnPt(CN)_6_.
