# Supplementary material for: Local Structure and Dynamics in MPt(CN)6 Prussian Blue Analogues
Source: Chem Mater. 2024 May 30;36(11):5796–804. doi: 10.1021/acs.chemmater.4c01013 (PMC11170939; doi:10.1021/acs.chemmater.4c01013)
Supplement: Supplementary file 1 — cm4c01013_si_001.pdf [file cm4c01013_si_001.pdf]

Local structure and dynamics in $MPt(CN)_6$ Prussian blue analogues

SUPPLEMENTARY INFORMATION

Elodie A. Harbourne,[1] Helena Barker,[1] Quentin Guéroult,[1]
John Cattermull,[1,2] Liam A. V. Nagle-Cocco,[3] Nikolaj Roth,[1]
John S. O. Evans,[4] David A. Keen,[5] and Andrew L. Goodwin[1,*]

[1]Department of Chemistry, University of Oxford, Inorganic Chemistry Laboratory,
South Parks Road, Oxford OX1 3QR, U.K.
[2]Department of Materials, University of Oxford, Parks Road, Oxford OX1 3PH, U.K.
[3]Cavendish Laboratory, University of Cambridge, JJ Thompson Avenue,
Cambridge CB3 0HE, U.K.
[4]Department of Chemistry, Durham University, Durham DH1 3LE, U.K.
[5]ISIS Facility, Rutherford Appleton Laboratory, Harwell Campus,
Didcot OX11 0QX, U.K.
*E-mail: andrew.goodwin@chem.ox.ac.uk.

Submitted to Chemistry of Materials

Contents

1 Iterative refinement of empirical parameters 3

2 Refinement results 10

3 CuPt(CN)$_6$ Phonon dispersion relations 15

4 References 17

1 Iterative refinement of empirical parameters

Overview

We provide in Fig. S1 a schematic illustrating our MC-driven constrained big-box modelling approach for producing atomistic representations of PBAs by refining against both X-ray PDF and conventional powder XRD patterns. The steps taken are as follows. (1) Our starting point is a knowledge of the basic average structure of $MPt(CN)_6$. This structure is then decorated with effective harmonic potentials, as described by Eq. (2) of the main text. In the first instance, the values of the various empirical parameters are initialised using sensible guesses. (2) A supercell configuration of the known average structure is then allowed to reach equilibrium under the action of these empirical potentials, using a Monte Carlo (MC) simulation running at an internal temperature that is eventually rescaled to 300 K (*i.e.* placing all empirical parameters on an absolute scale). The resulting configuration captures both local structure information—in the form of individual atomic displacements—and average structure information—in the form of the atom distributions obtained by projection onto the parent cell. (3) Using TOPAS, the Bragg diffraction pattern and PDF are calculated from the MC model and additional fitting parameters are allowed to refine. The goodness-of-fit R_{wp} is then recorded. Empirical parameters are then modified one-by-one and the cycle is repeated, allowing optimisation of each parameter to produce the best fit to data.

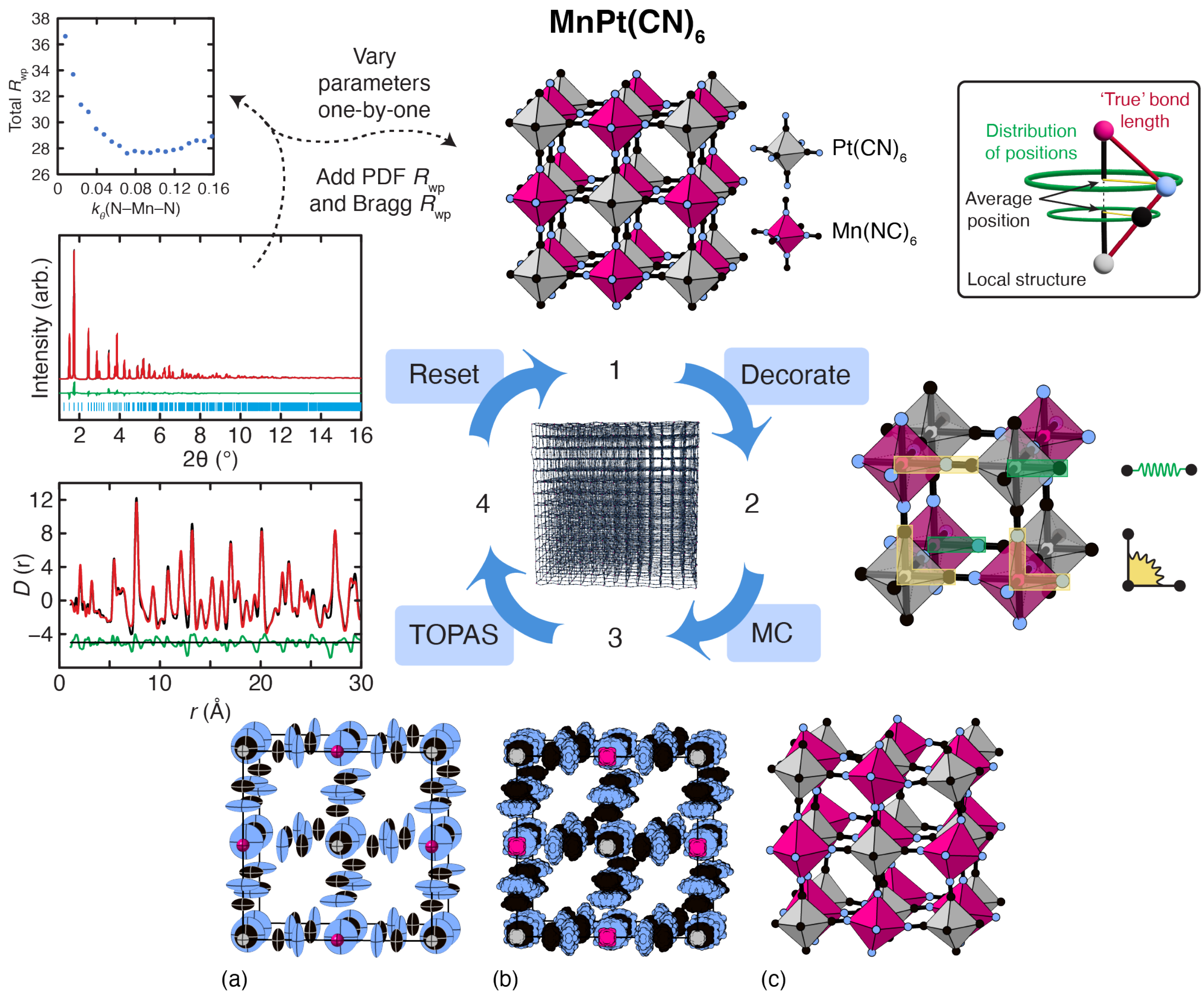

Figure S1: Overview of the emprical parameter optimisation strategy employed in our study. The inset in the top-right corner shows a representation of a transverse vibrational mode of cyanide bridging atoms;[S1] the 'apparent' polyhedral bond lengths are shown in black, which are inferred from the distance between the average positions of two atoms, as determined from Bragg analysis; the 'true' bond lengths are shown in red, which are the average distance between two atoms.

Initial Pawley refinements

As a preliminary step in our analysis, we performed a conventional Pawley refinement of the $MPt(CN)_6$ X-ray powder diffraction patterns over the angular range $1 \leq 2\theta \leq 16^\circ$. These fits allowed us to determine a zero error, an axial divergence parameter, and unit-cell dimensions. The zero error and axial parameter were fixed for subsequent TOPAS refinements, and the unit-cell parameters were used in our Monte Carlo simulations.

Direct Monte Carlo simulations

MC simulations were carried out using a custom code written in Fortran90. We employed $6 \times 6 \times 6$ supercells of the parent face-centred cubic $MPt(CN)_6$ supercell, such that each configuration contained 12 096 atoms in total. We fixed the MC supercell dimensions according to the unit-cell parameters determined during our preliminary Pawley refinement of the powder X-ray diffraction data. The coordinates of M, Pt, and N atoms were treated as free variables; the C atom positions were determined by decorating the corresponding Pt–N vectors. In this way, neither the C–N bond length nor the Pt–C–N angle were allowed to deviate from their ideal values. The MC energy was determined according to the expression

$$E_{\mathrm{MC}} = \frac{1}{2}\sum_{\mathrm{bonds}} k_r(r - r_{\mathsf{e}})^2 + \frac{1}{2}\sum_{\mathrm{angles}} k_\theta(\theta - \theta_{\mathsf{e}})^2. \quad (1)$$

Here, r_{e} is the ideal length of a given bond, θ_{e} is the ideal angle for a given triplet, and k_r and k_θ are force constants. An atom was selected at random, moved by a small amount, and the move accepted or rejected according to the usual Metropolis algorithm. This process was repeated until convergence (roughly 150 accepted moves / atom), which took approximately 3 mins when running on a standard laptop computer. The resulting configuration was then used to output a TOPAS file for subsequent comparison against experimental X-ray scattering data. This file includes two representations of the MC configuration: the explicit atom coordinates are used for calculation of the PDF, and a projection of the coordinates onto the parent cell is used for calculation of the Bragg intensities.

Atomistic TOPAS refinements

Our TOPAS refinements consisted of two main blocks: the first corresponding to the calculation of X-ray Bragg intensities from the (projected) average structure of our MC simulations, and the second corresponding to the calculation of the X-ray PDF from the individual atom coordinates within the MC configurations.

For the Bragg scattering component, we allowed free refinement of unit cell parameters, scale, a background function, and the parameters of a Thompson-Cox-Hastings pseudo-Voigt (TCHZ) peak shape function. Fits were again performed over the angular range $1 \leq 2\theta \leq 16^{\circ}$. The refinements were carried out using $P1$ crystal symmetry with the cell metric constrained appropriately (cubic for M = Mn and tetragonal for M = Cu). This approach is equivalent to applying space-group symmetry operations to the projected coordinates obtained from MC and subsequent Rietveld refinement in the corresponding space group setting. Relative atom coordinates were fixed, and each atom site was assigned a (fixed) isotropic displacement parameter $B_{\text{eq}} = 0.1$ Å^2, which was chosen to be very much smaller than the width of the atom distributions and also the real-space resolution of the X-ray diffraction pattern.

For the PDF component, we used a fixed Q-damping term of $\mathrm{d}Q = 0.08$ Å^{-1}, a fixed $Q_{\max}$ convolution term of 19^{-1} Å, and convolved our calculations with an r-dependent broadening term (α) which was allowed to refine freely. Unit-cell parameters were coupled to those refined in the Bragg scattering component, taking into account the additional factor associated with the supercell size. Fits were performed over the range $1 < r < 30$ Å, with data binned at intervals of 0.02 Å. Atom positions were again fixed (now within the larger MC supercell) and each atom was assigned the same small (fixed) isotropic displacement parameter $B_{\text{eq}} = 0.1$ Å^2.

The only parameters shared between Bragg and PDF components were the unit-cell parameters, to which the Bragg component has a much greater sensitivity. Accordingly, the PDF fits were included in the least-squares cycles using a relative weighting of 0.001, which ensured that the cell parameter fitting was dominated by the Bragg component. This weighting does not affect the refined values of any remaining parameters. The quality of fit was monitored independently for both Bragg and PDF components through the corresponding R_{wp} values, with final refinements converging to values of about 8% and 20%, respectively.

Parameter search

Our starting point is a set of parameters $\mathcal{P}_0 = \{k_r, k_\theta, r_\mathrm{e}\}$, whose initial values are guessed from reasonable assumptions (*e.g.* average-structure bond lengths). We use the subscript '0' to denote initial values. Our parameter search then proceeds in cycles. For the ith cycle, the goodness-of-fit is determined by taking the set $\mathcal{P}_{i-1}$ and then varying in turn each parameter within this set by small increments. These variations are carried out independently of one another in the sense that, as one parameter is varied, the others are kept constant at the values in $\mathcal{P}_{i-1}$. In this way, we are effectively mapping the fit-quality landscape in section along each of the variables. Having carried out this search, the instance that corresponds to the highest quality fit-to-data is identified, and the subsequent parameter set $\mathcal{P}_i$ formed by updating the single relevant variable to its new value. Hence parameter sets in subsequent cycles differ only by a single parameter. We continue from cycle to cycle until no further improvement in quality of fit can be obtained.

We show in Figs. S2 and S3 representative traces of the goodness-of-fit for variations in (respectively) the equilibrium M–N bond lengths and M–N bond-stretching parameters obtained in the final parameter search cycle for both $MnPt(CN)_6$ and $CuPt(CN)_6$. An important consideration to keep in mind is that varying r_e(M–N) does not correspond to varying the position of the maximum in $g_{\text{M–N}}(r)$, but instead affects in a complex manner the interplay of different bond-lengths in the evaluation of the MC energy via Eq. (1). Hence uncertainties in these parameters must be interpreted differently to uncertainties in the final partial pair distribution functions. A point made obvious by these figures is that the PDF and Bragg components often drive the model in opposite directions; this tension is well-known in the RMC field, for example.[S2] In the absence of a strict rule for weighting the PDF and Bragg components, we have used the simple combined fit to discriminate models.

Discussion

While other parameter search mechanisms can easily be envisaged, we note that the approach we take here operates by optimising first those parameters to which the data has greatest sensitivity. The search process is itself deterministic and so can easily be automated, albeit that small fluctuations in the values of R_wp arise from the stochastic nature of the MC simulations

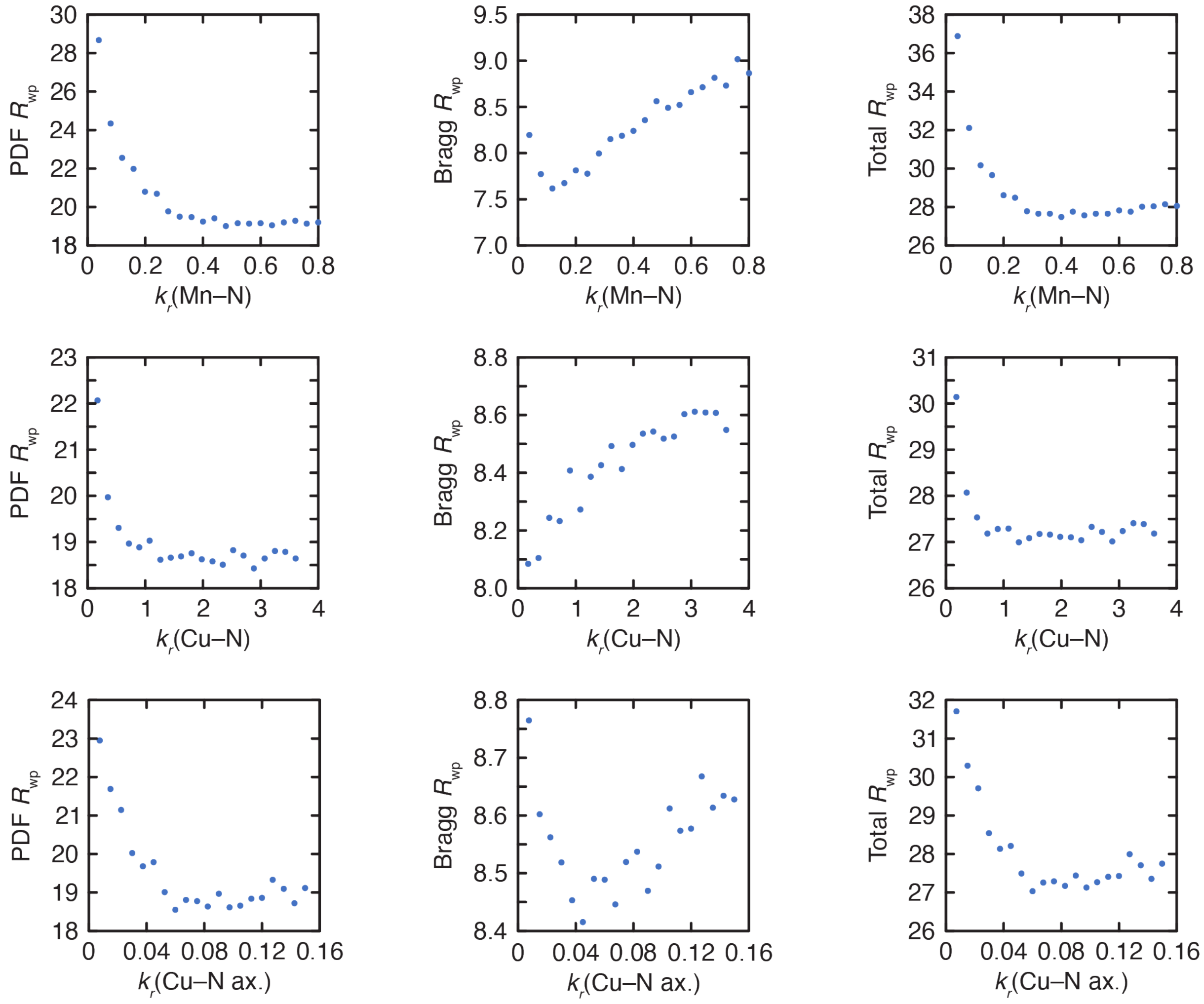

Figure S2: Goodness-of-fit as a function of k_r(M–N).

from which the corresponding fits are derived. There is also no guarantee that the methodology arrives at the global minimum and a goal for future work would be to automate the parameter search algorithm using more sophisticated approaches better suited to multi-dimensional landscapes (*e.g.* genetic algorithms). It would also be desirable to extract quantitative measures of the uncertainty in each variable and the corresponding covariances. For the purposes of this study, however, we are content that the final parameter sets obtained provide fit qualities that are comparable to those of conventional PDF (and Rietveld) refinements. Indeed the fit qualities are comparable to those obtained using fully-atomistic refinements (*e.g.* RMC), despite the

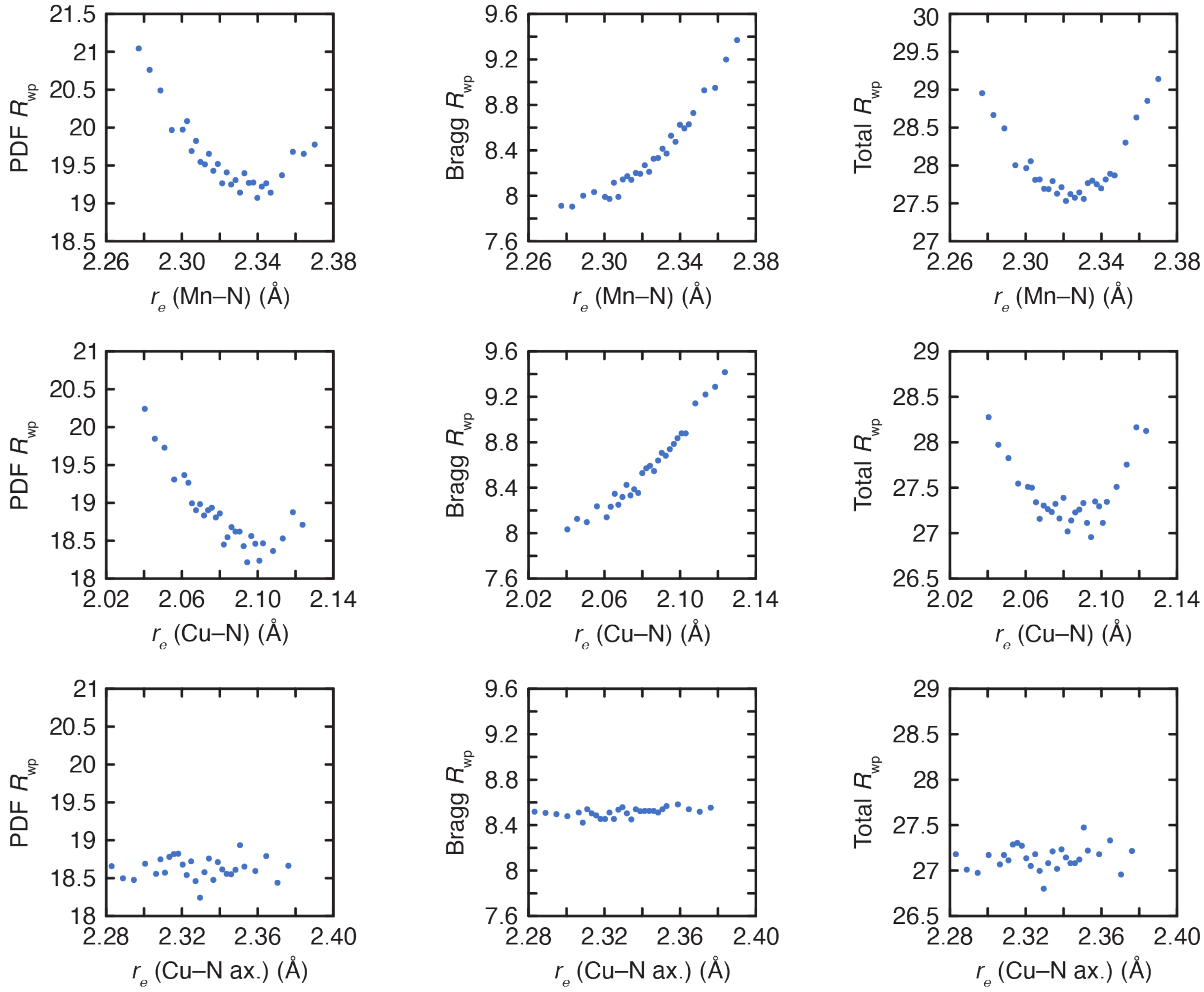

Figure S3: Goodness-of-fit as a function of r_e(M–N).

vast reduction in number of free parameters ($\mathcal{O}(10^1)$ *vs* $\mathcal{O}(10^5)$). Nevertheless, we make no claim that the final parameter sets represent a unique description of the X-ray scattering data.

2 Refinement results

The final fits-to-data are given in the Fig. 2 of the main paper and the corresponding parameter values are listed in Table 1 of the main paper. We include in Fig. S4 and S5 cross-sections of representative MC simulations generated using the final parameters for both MnPt(CN)$_6$ and CuPt(CN)$_6$. Crystallographic details of the average structures formed by projections of these MC configurations are described in Tables S1 and S2. For the case of CuPt(CN)$_6$ we give our results in terms of the standard space-group setting $I4/mmm$, but note that our refinements were carried out using the non-standard $F4/mmm$ setting as described in the text. Representations of the average structure of MnPt(CN)$_6$ and CuPt(CN)$_6$, showing anisotropic displacement parameters as ellipsoids, are given in Fig. S6.

Table S1: Crystallographic parameters for the structure of MnPt(CN)$_6$ at ambient temperature and pressure. The experimental data were collected at I15-1 beamline, Diamond Light Source, using a wavelength of $\lambda = 0.1617$ Å.

Space group	$Fm\bar{3}m$								
a/Å	10.74419(11)								
V/Å^3	1240.28(4)								
Z	4								
R_{wp}/% (Bragg)	8.31								
R_{wp}/% (PDF)	19.14								
Atom	x	y	z	U_{11}/Å^2	U_{22}/Å^2	U_{33}/Å^2	U_{23}/Å^2	U_{13}/Å^2	U_{12}/Å^2
Pt	0	0	0	0.00961(9)	0.00961	0.00961	0	0	0
Mn	0.5	0	0	0.01118(11)	0.01118	0.01118	0	0	0
C	0.18642(7)	0	0	0.01016(10)	0.0417(3)	0.0417	0	0	0
N	0.29122(7)	0	0	0.01029(10)	0.0884(6)	0.0884	0	0	0

Table S2: Crystallographic parameters for the structure of $CuPt(CN)_6$ at ambient temperature and pressure. The experimental data were collected at I15-1 beamline, Diamond Light Source, using a wavelength of $\lambda = 0.1617$ Å.

Space group	$I4/mmm$								
a/Å	7.27638(11)								
c/Å	11.0245(3)								
V/Å^3	583.70(2)								
Z	2								
R_{wp}/% (Bragg)	8.31								
R_{wp}/% (PDF)	19.14								
Atom	x	y	z	U_{11}/Å^2	U_{22}/Å^2	U_{33}/Å^2	U_{23}/Å^2	U_{13}/Å^2	U_{12}/Å^2
Pt	0	0	0	0.00784(9)	0.00784	0.0196(3)	0	0	0
Cu	0.5	0.5	0	0.00822(10)	0.00822	0.0211(4)	0	0	0
C1	0.19091(7)	0.19091	0	0.0229(2)	0.0229	0.0435(5)	0	0	−0.0151(2)
N1	0.30286(7)	0.30286	0	0.0466(5)	0.0466	0.0840(9)	0	0	−0.0388(5)
C2	0	0	0.18109(16)	0.0340(4)	0.0340	0.0210(4)	0	0	0
N2	0	0	0.28569(16)	0.0745(8)	0.0745	0.0211(4)	0	0	0

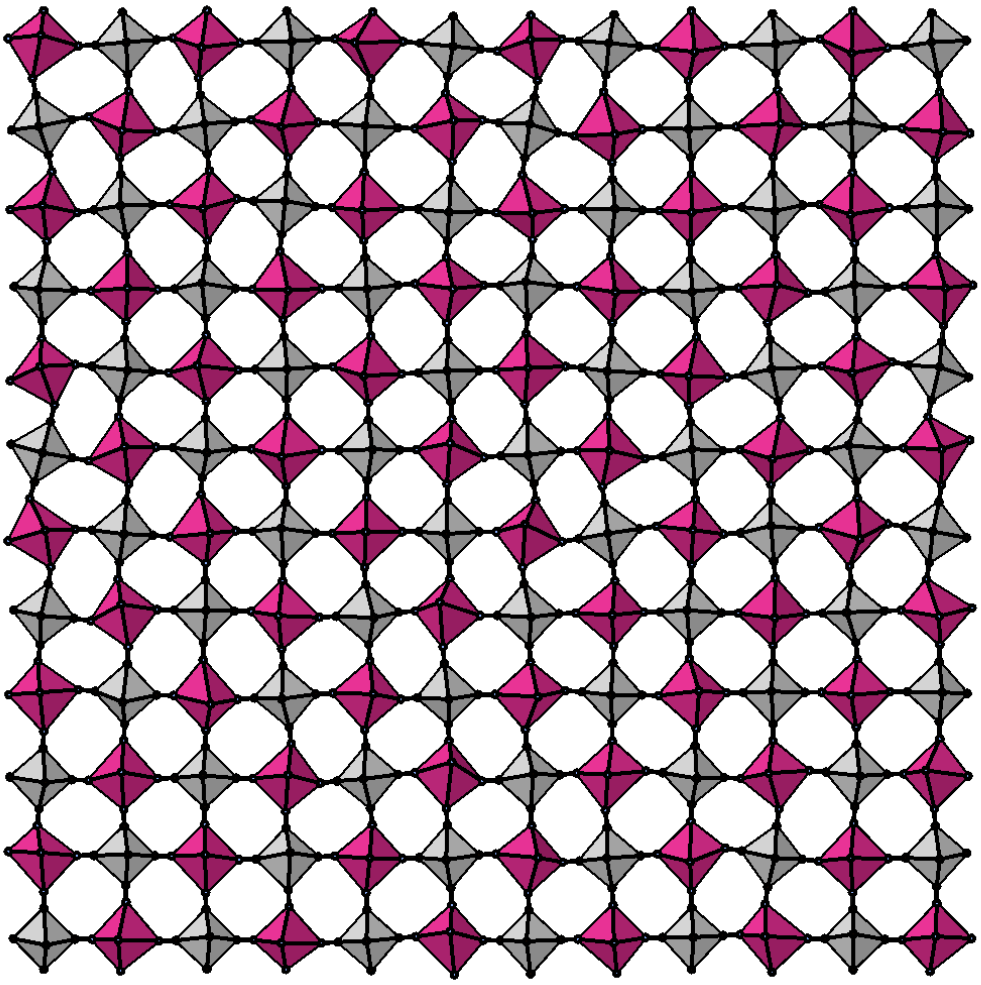

Figure S4: Cross-section of a representative $MnPt(CN)_6$ configuration.

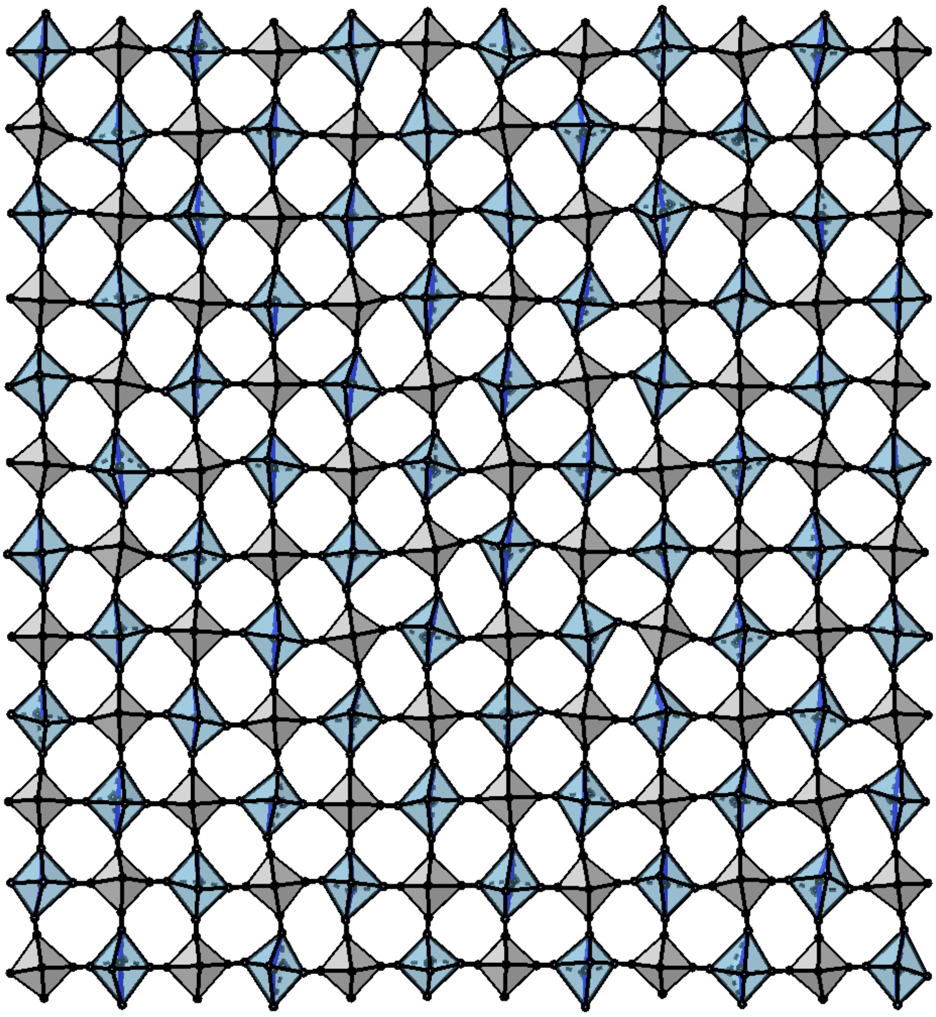

Figure S5: Cross-section of a representative $CuPt(CN)_6$ configuration.

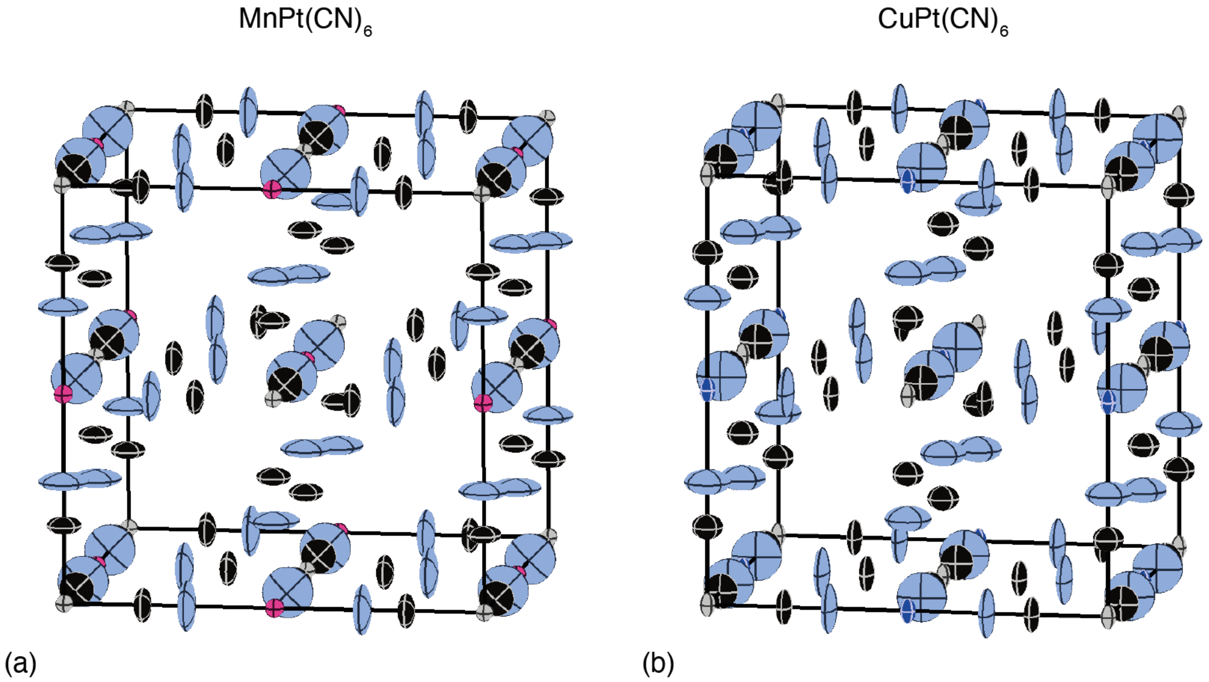

Figure S6: Representations of the average structure of (a) $MnPt(CN)_6$ and (b) $CuPt(CN)_6$, determined from projections of representative MC simulations. Atomic displacement ellipsoids are shown at 80% probability.

3 $CuPt(CN)_6$ Phonon dispersion relations

The phonon dispersion relations for $CuPt(CN)_6$, calculated from our empirical model, are shown in Fig. S7. While the phonon spectrum spans similar energies to that of $MnPt(CN)_6$ shown in Fig. 5(a) of the main text, there are a number of key differences that deserve comment. The primary difference arises because the reduced crystal symmetry of $CuPt(CN)_6$ associated with cooperative Jahn–Teller distortion increases the number of symmetry-distinct phonon branches, and also breaks the degeneracy between the two X points (we retain the face-centred cubic nomenclature for high-symmetry points in the Brillouin zone). The tilt modes are nonetheless retained at approximately 10 meV, and again these are associated with large and negative Grüneisen parameters. Interestingly, a non-rotational branch of the phonon dispersion has fallen to a similar energy scale in this case. These non-rotational phonons appear to involve deformations within the CuN_6 octahedra and arise from the weaker potentials involving axial Cu–N linkages. Because the bending of Cu–NC–Pt linkages behaves like the transverse modes of the 'tension mechanism' for NTE, this branch in the phonon spectrum is also associated with strongly negative Grüneisen parameters.

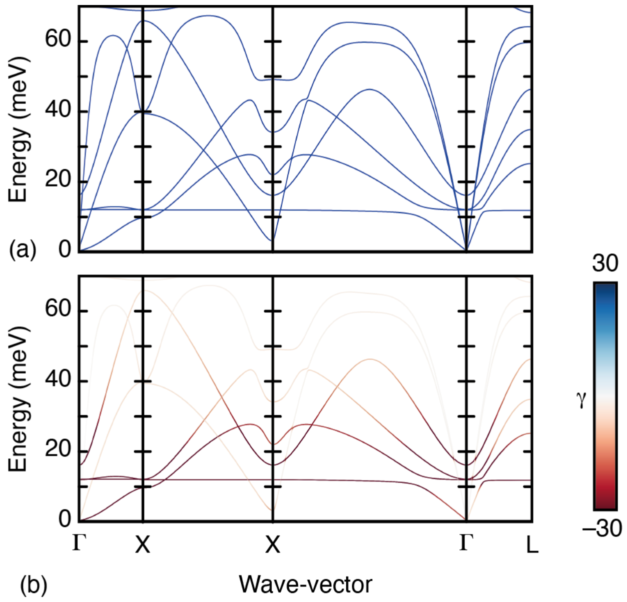

Figure S7: Phonon dispersion relations for $CuPt(CN)_6$ determined from our PDF-derived lattice dynamical model. Panel (a) shows the phonon dispersion itself, and panel (b) shows the same set of curves, coloured according to the corresponding mode Grüneisen parameters.

4 References

(S1) Chapman, K. W., Chupas, P. J. & Kepert, C. J. Direct observation of a transverse vibrational mechanism for negative thermal expansion in $Zn(CN)_2$: An atomic pair distribution function analysis. *J. Am. Chem. Soc.* **127**, 15630–15636 (2005).

(S2) Playford, H. Y., Owen, L. R., Levin, I. & Tucker, M. G. New insights into complex materials using reverse Monte Carlo modeling. *Ann. Rev. Mater. Res.* **44**, 429–449 (2014).
